# “It’s Like Doctor Jekyll and Mister Hyde”: The Construction of Moral Identity by Israeli Men Who Pay Women for Sex

**DOI:** 10.1007/s10508-024-03073-3

**Published:** 2025-01-13

**Authors:** Ayelet Prior, Einat Peled

**Affiliations:** The Bob Shapell School of Social Work, Tel Aviv University, Ramat Aviv, P.O.B. 39040, 6997801 Tel Aviv, Israel

**Keywords:** Men who pay for sex, Sex for pay, Identity construction, Moral identity, Moral reflexivity, Sex work

## Abstract

This study examined how Israeli men who pay women for sex (MPWS) construct and sustain a moral identity within the social context that often portrays them as deviants, perpetrators, and abusers, thereby challenging their ability to maintain a respectful and dignified image. Twenty-three Israeli MPWS participated in in-depth semi-structured interviews, which were then analyzed using constructivist grounded theory method. Using the theoretical framework moral reflexivity, we conceptualize three central dynamics of constructing and sustaining a moral identity in the context of paying for sex: Maintaining an intact moral self by resisting the moral conflict; presenting a reflexive agonizing moral self; and constructing a moral self through identity fragmentation. The findings reveal that these facets of the moral reflexivity process can overlap, coexist, and circulate in a messy manner, thus promoting an ecological understanding of how morality is shaped by various societal forces, rather than discovering what a moral identity is. We therefore call for a morally sensitive approach in studies in the field of MPWS and sex work. Such an approach encourages researchers to be aware of moral issues, moral questions, and moral processes, and to treat morality as a socially context-dependent trait that is highly relevant to the study of the sex industry.

## Introduction

The debate over the sex industry and the commerce of sex is often a moral one (Agustin, 2008; Östergren, 2017), as the commodification of bodily attributes carries ethical difficulties, and the exchange of sex for money is steeped in moral significance (Bernstein, 2007; Weitzer, 2018). Across diverse social contexts and under varied regimes, selling sex and paying for sex are stigmatized, and the people who are involved in the sex industry are considered demeaned, tainted, and morally damaged (Benoit et al., 2018; Weitzer, 2018). An increasingly popular moral frame portrays men who pay women for sex (MPWS) as deviants, perpetrators, and abusers (Peled et al., 2020; Weitzer, 2009), and consequently they struggle to maintain a respectful and dignified image. Accordingly, the present study focuses on exploring how Israeli MPWS construct and sustain a moral identity, within the particular stigmatizing Israeli context that portrays them as having a “spoiled identity” (Goffman, 1963)—i.e., deviant—as described below.

### The Moral Nature of the Debate Over Sex Trading

The sex industry is frequently viewed as a quintessential example of a market exchange imbued with moral implications. Historical and ongoing moral discourses deeply impact public, professional, and academic discussions about sex trading (Bernstein, 2007). Global debates, research, and policy surrounding the sex trade are often driven by explicit moral ideologies, and consequently they are criticized for being emotionally charged rather than empirically grounded (Wagenaar & Altink, 2012). Traditionally, these discussions depict individuals in the sex trade as threats to societal ethics, othering them as criminals, deviants, and “dirty” (Abel & Fitzgerald, 2010). People in the sex trade, particularly women who are paid for sex (WPS), are often portrayed as troubled, blameworthy, and are labeled as morally flawed sexual deviants (Macias-Konstantopoulos & Bar-Halpern, 2016). In recent decades, there has been a shift in these moral narratives. Instead of solely labeling those who sell sex as deviants, the commodification of sex itself is increasingly seen as inherently morally unacceptable. Moral condemnation now often focuses on the purchasers of sex, casting them as morally defective and abusive perpetrators, and depicting WPS as helpless victims needing protection (Abel, 2014; Vanwesenbeeck, 2017).

In the Israeli context, moral discourses and norms regarding sex trading are mostly shaped by conservative perceptions, liberal secular perceptions, and feminist perspectives (Lavee & Katz, 2003; Okun, 2016; Shamir, 2018). The conservative discourse, which is strongly influenced by Jewish orthodox religious beliefs, depicts prostitution^1^ as a moral problem. Prostitution and holiness are described as opposites, with prostitution seen as obscenity and as the antithesis of the holiness required of a Jewish person (Zoldan, 2006). The demand to abstain from prostitution extends not only to the women involved in it, but also to those who seek their services. Therefore, from a conservative perspective, MWPS are constructed as individuals who are captivated in lust and depravity, willing to trample on the dignity and soul of those in prostitution to satisfy their desires (Levy, 2020).

The liberal approach frames sex trading as a matter of individual choice that should generally remain free from regulation (Shamir, 2018). From this perspective, sex trading is viewed as a free-market transaction between two (or more) consenting adults acting as equal negotiators (Amir & Amir, 2004), with no moral judgment attached to their actions. Within this discourse, zones of tolerance for sex trading are presented as ideal spaces where such transactions can occur safely, without criminalization or condemnation of any party involved (Shamir, 2018).

The radical feminist discourse that paints paying for sex as a form of violence against women has become increasingly prevalent in the past decades in the Israeli public, professional, and social advocacy discourses (Peled et al., 2020). Radical feminism frames any form of sex trading as sexual exploitation and sex trafficking, thus depicting sex for pay (SFP) as a felony (Ekberg, 2004). The growing influence of this viewpoint is evident in the rise of anti-trafficking and end-demand movements (Abel, 2014). Indeed, in 2018, the Israeli parliament passed the Prohibition of Prostitution Consumption Law, which came into effect in July 2020, following a public campaign led by radical feminist activists in conjunction with conservative and Jewish Orthodox political figures (Levy-Aronovic et al., 2021). This law criminalizes the purchase of sexual services, subjecting offenders, i.e., Israeli MPWS, to either an administrative fine or the requirement to attend an educational program.

Notwithstanding the growing popularity of the radical feminist discourse among professionals and policymakers, a recent study on Israeli public attitudes toward the End Demand legislation found that the majority of the Israeli public have not formed an opinion regarding the effectiveness of this legislation (Shamir et al., 2024). The researchers suggest that this may indicate a general disinterest in the well-being of WPS, possibly because they are perceived as morally degraded. Furthermore, this study shows that among supporters of the End Demand legislation in Israel, support for criminalizing WPS has also increased (Shamir et al., 2024). This demonstrates that as support for End Demand legislation grows, the general public’s attitude increasingly focuses on repressive measures that view WPS as morally flawed (Grönvall, 2023).

Thus, the sociopolitical and cultural perceptions regarding the morality of sex trading in Israel are layered, as they usually depict it as a moral felony committed by WPS and/or MWPS. Either way, we presume that the moral identity of MPWS is deeply entrenched in Israel’s social, cultural, legal, and religious constructions of sex trading. Accordingly, the present study focuses on understanding how Israeli MPWS construct and negotiate their moral identity within these particular discursive contexts.

### The Morality of Men Who Pay Women for Sex

Previous studies discussed the moral difficulties that MPWS usually encounter, and the various strategies that they apply to portray themselves as normative or non-deviant (Lahav-Raz et al., 2024). Studies have suggested, for example, that MPWS feel morally flawed for betraying their partners (Horswill & Weitzer, 2018); worry about the well-being of WPS; hate seeing themselves as abusers (Huschke & Schubotz, 2016); are ill at ease for breaking the law and performing criminal acts (Sterling et al., 2018); and feel guilty for flouting religious norms that forbid SFP (Chen, 2017).

MPWS apply various strategies to deal with the moral difficulties they encounter. These include resorting to consumerist notions or masculine repertoires to discuss their engagement in SFP as part of the normative realm of market exchange (Lahav-Raz, 2019; Pettinger, 2013) or masculine biological needs (Bounds et al., 2017); joining online communities of MPWS that offer supportive experiences of brotherhood (Lahav-Raz, 2020) and ease the sense of stigmatization and social labeling (Hammond, 2015); condemning other (abusive and violent) MPWS in a bid to differentiate themselves from such despicable image (Chu & Laidler, 2016; Sterling et al., 2018); and traveling abroad to pay for sex, to avoid the moral taint directed at them in their home countries (Lahav-Raz et al., 2024; Prior & Peled, 2019).

The findings about the morality of MPWS suggest that it is simultaneously shaped by global and local sociocultural moral norms regarding sex trading, as well as personal characteristics and attributes of individual MPWS. Therefore, the theoretical concept of moral identity is highly relevant. A constructivist conceptualization of moral identity advances the understanding of how societal constructs of morality are interpreted by individual MPWS and translated into their everyday moral practice (Charmaz et al., 2019). Namely, by applying the concept of moral identity, we aspired to deepen the understanding of the unique and particular dynamics and processes that Israeli MPWS pursue in an effort to construct and maintain their morality in the face of social, religious and situational critic and stigma.

### The Theoretical Perspective of Moral Identity

Moral identity is typically defined as the extent to which morality is reflected as being essential or central to the individual’s sense of self (Blasi, 1983). From a cognitive developmental standpoint, moral identity is usually the product of the emergence of moral reasoning in childhood and adolescence (Hardy & Carlo, 2011b). Cognitive development theories adopt an essentialist stance to moral identity, as they view it as being founded on the development of the capacity to judge right from wrong, and good from bad (Hardy & Carlo, 2011a). The most popular theoretical model in this field suggests that moral identity bridges between moral judgment and moral action—namely, it is the sense of responsibility that one feels to act upon one’s moral judgment (Blasi, 1983). The cognitive developmental model also suggests that individuals aspire for a consistent sense of self, so moral responsibility or moral identity reflect the centrality of morality in one’s sense of identity (Hardy & Carlo, 2011b).

Constructivist perspectives suggest that morality is often seen as the equivalent of “good,” and a moral person is one who understands the difference between good and bad, right and wrong, worthy and unworthy, just and unjust (Hitlin & Vaisey, 2013). This view assumes that the definitions of good and bad are socially embedded, and therefore moral identity is not a fixed category but a fluid and contextual construct (Lukes, 2010). Thus, instead of portraying moral identity through traits such as honesty, altruism, or care for others (however conventional these might appear), morality is defined as a set of cultural collective codes of an evaluative and obligatory character that specifies what is good and bad, responsible and irresponsible, or just and unjust (Shadnam, 2020). Moral identity is, therefore, rooted in sociocultural norms, and signifies how important it is for the individual to be good and to do the right things in a given social context.

The constructivist perspective further suggests that individuals in a given society or social group are always situated in a matrix of existing moral norms, which they often adopt in an automatic, intuitive and subconscious fashion (Shadnam, 2015). In this regard, the process of constructing a moral identity is described as nonreflexive (Zigon, 2007), and involves adopting the sociocultural moral norms of the society one lives in as self-evident truths. But in situations where individuals pause and ponder about a given moral norm, they become aware of their position about that norm, and reflect upon their own moral self-narrative in relation to it (Shadnam, 2020). Shadnam called this process moral reflexivity, and suggested that it is a process of questioning and rethinking “Who am I?” in light of the sociocultural categories that are associated with a given moral norm.

Moral reflexivity therefore occurs when an individual’s moral self-narrative is challenged. It is a process involving “how individuals view and evaluate themselves, how they define who they really are as persons, and how they understand their authentic selves” with regard to a given moral norm (Shadnam, 2020)—a self-reflexive process of choosing who we want to be. This reflexive process is triggered by critical moments (Boltanski & Thévenot, 1999) of questioning a moral norm—namely, the emergence of doubts about it—that can lead to a moral breakdown (Zigon, 2007)*,* in which the individual is abruptly torn from their nonreflexive and somewhat self-evident moral being into a process of reinventing or reconstructing their moral self. The reinvention of one’s moral self does not necessarily imply a change in behavior. It might very well be that an individual might return to a nonreflexive stance. Nonetheless, a somewhat circular movement occurs as one reflects on their morality, reevaluate their self accordingly, and reorganizes their moral self according to their evaluation.

The reproduction of a moral self is integral to one’s existing self-concept (Shadnam, 2020), and thus always conditioned by one’s available symbolic resources. These resources are the tools with which one can reflect upon, and subsequently reconstruct, one’s moral self. For individuals seeking to understand who they are with respect to a given moral norm, symbolic resources are the norms, discourses and social constructions that are the phantoms of possible answers. These constructions serve as thumbnails that one may browse, combine, and modify, in a bid to reform one’s moral self. As Shadnam (2020) explained: “No moral reflection starts from scratch, and any individual who is in a quest to choose whom to be is always provided with a number of identities related to the moral norms at issue” (p.17).

In the present study, we surmised that MWPS may experience various critical moments that trigger their process of moral reflexivity with regard to the multiple moral norms surrounding sex trading and SFP. Specifically, we were interested in understanding how Israeli MPWS use (and reject) religious and sociocultural symbolic resources to maintain a moral image in a context that mostly constructs their behavior as damaged and flawed. Furthermore, we sought to uncover the moral reflexivity of MPWS as it was constructed through verbal and symbolic communication, that in turn reflect relevant social discourses (Charmaz et al., 2019), within a setting of a conversation with a female interviewer whom they usually assumed would be judgmental toward them.

## Method

The interpretive epistemology guiding this qualitative study posits that knowledge is interactively constructed throughout the research process within particular temporal, relational and spatial contexts (Guba & Lincoln, 1998). Accordingly, this study employed a constructivist grounded theory method, by analyzing in-depth semi-structured interviews with Israeli MWPS, with a view to capturing the multilayered identity-related meanings that the men assigned to their experiences in SFP (Charmaz, 2014).

### Participants

A total of 23 Israeli adult MWPS took part in the study. They were recruited by several means: (1) Facebook ads posted on groups dealing with sex work, sex, gender, and intimacy and the authors’ private profiles; (2) therapists specializing in treating sex addiction and sexual dysfunction; (3) personal acquaintance; and (4) snowball sampling. Thirty-five men responded to the initial invitation, 12 of whom eventually withdrew, mostly for fear of being exposed, or because they had mistaken the research appeal as an advertisement of sexual services.

The average age of the participants was 39.6 years (range = 22–72). Eighteen participants were born in Israel, three in the former USSR, one in Turkey, and one in the USA. Seventeen participants were of Jewish secular background, four were of Jewish religious background, and two were of Jewish “Haredi” (ultra-religious) background. Twelve participants were high-school graduates, ten were university educated, and one had dropped out after elementary school. Fifteen participants worked at white-collar jobs, four at blue-collar jobs, two were students, and two were unemployed. Nine participants were single, eight were married, and six were divorced. Thirteen participants had children.

In terms of SFP habits, nine participants had paid for sex over a period of between 1 and 10 years, five over 11–20 years, and eight for over 21 years (one participant declined to say). Three participants declared that they no longer pay for sex, while the remainder were still actively involved in sex-purchasing at the time of the interview. The participants’ SFP experiences were in diverse settings—including street-based, parlors, hotels, private home, and strip clubs.

### Measures and Procedure

The 23 participants engaged in 28 in-depth semi-structured interviews. (The disparity in numbers is because in four instances of an incomprehensive interview, the participant was invited to a follow-up interview; three participants had two consecutive interviews, and one had three more interviews.) All interviews were conducted by the first author in the course of 2019–2020, when the legal status of MPWS had already changed, but the criminalization was not yet enforced. Hence, it is within this threatening and stigmatizing context that the data were collected and later analyzed.

The interviews usually lasted about 1.5 h (range: 30 min to 2.5 h). At the participant’s request in each case, six interviews were conducted by telephone or Skype, and 22 in person at locations such as coffee shops that enabled relative privacy, or in private offices. The interview protocol comprised the following topics: the interviewee’s personal experience in SFP; his interactions with WPS and with other MWPS; his perceptions of masculinity, consumerism, normativity, deviancy and morality (in the context of SFP); and his identity dilemmas related to his experience in SFP.

### Data Analysis

Data analysis was led by the first author under the supervision of the second author, in accordance with constructivist grounded theory procedures (Charmaz, 2014), using MAXQDA 2022 qualitative analysis software. This was begun after the first few interviews and continued throughout and after data collection, with consequent updates to the interview protocol to reflect new understandings. Thus, for example, after initial coding of the first few interviews, we added a question about conflicts and contradictions in the participants’ experiences of SFP, as this was found to be relevant to understanding identity dilemmas and practices of identity work. When the coding process reached saturation and no longer yielded new insights, we ended the data collection, and the interviews were coded into several categories of prominent identities in the participants’ narratives—one of them being the moral identity. Next, we delved into the literature of morality and moral identity, and identified the concept of moral reflexivity (Shadnam, 2020) as being useful for capturing how MPWS construct and maintain a moral identity.

#### Positionality and Ethical Consideration

Our extensive experience in studying MWPS from a constructivist approach has taught us that SFP is an intricate social phenomenon. Accordingly, we reject binary definitions of the impact and meaning of SFP, and are committed to interpreting the participants’ narratives with all their complexities and contradictions. Our familiarity with this population and with the phenomenon, and our experience in interviewing MWPS, made it possible to foster a relaxed atmosphere in most interviews, and the participants’ sharing of explicit, rich and complex sexual and intimate narratives of SFP.

Nonetheless, the intense and dynamic sociolegal context in which the study took place was evident in some of the interviews. Most of the participants presumed that the interviewer was a staunch supporter of the new Prohibition on Prostitution Consumption Law, and many were motivated to take part in the study in the hope that the findings would prove that the new law is misguided, and perhaps even prevent its implementation. Thus, some of the interviews were somewhat tense (Prior & Peled, 2022)—particularly when the participants discussed moral questions and attempted to resist the social labeling that they assumed the interviewer agreed with.

All participants signed consent forms. The participants were free to end their participation at any time, or refuse to answer certain questions without any adverse consequences, and a debriefing conversation was held at the end of each interview. Most participants remarked that the interview atmosphere was nonjudgmental and respectful, and were pleased to have had the opportunity to discuss their experiences. Participants’ names and other identifying details have been changed or omitted to maintain confidentiality.

## Results

As previously noted, we used the concept of moral reflexivity to explore the processes of constructing a moral identity by Israeli MPWS. We identified three central dynamics of constructing and sustaining a moral identity in the context of SFP: Maintaining an intact moral self by resisting the moral conflicts; Presenting a reflexive, agonizing moral self; and Constructing a moral self through identity fragmentation. These dynamics were also apparent within the interviews, as evident below.

### Maintaining an Intact Moral Self by Resisting the Moral Conflicts

The first dynamic of moral self construction that we identified was that of maintaining a sense of an intact moral self, by resisting potential moral conflicts. Despite their awareness of the morally challenging context, some of the participants did not actually experience a moral conflict—i.e., appeared to feel no need to engage in moral reflexivity over their SFP behavior. Roman, for example, was well aware of the radical feminist moral criticism directed at MPWS for dehumanizing and objectifying WPS (Tyler & Jovanovski, 2018)—but was unwilling to give in to the objectification discourse, let alone engage in moral reflection about his own personal behavior. For him, SFP was a commercial transaction that needn’t be discussed from a moral standpoint:

Some people may call my approach materialistic—or how do they call it? “Objectification,” shmobjectification. Fine. Er, as long as one side wants to work and earn money, and the other side agrees with the terms, then all the criticism about objectification shmobjectification is completely irrelevant. It’s nobody’s business, it’s a personal matter, and as long as there is no violence or abuse, then everything is cool. I think that calling it “objectification” or “shmobjectification,” or whatever, or banning it because a person is being looked at as a product—fine. It’s a private matter between these two people, and it’s their business how they look at one another. [Roman]

Unlike Roman, other participants were preoccupied with the morality of the sex industry, yet even when they experienced critical moments in which their morality was questioned, it did not lead to moral reflexivity. Arieh, for example, discussed at length the morality of WPS throughout his interview expressing a perception that aligns with the whore stigma (Pheterson, 1993) as he explained that selling sex is morally problematic, and that women who engage in it are morally damaged:

These girls go out [to bars], alone, and you can sleep with them for nothing. So some of them say “If I’m going to sleep anyway with someone I like, who will buy me [a drink], I might as well get money for it” […] when your morality—or lack of it—when your standards are low, you get pulled into this thing of taking money [for sex]. And then you start working in it [Arieh].

Interviewer: Because this thing of selling sex is morally low?

Arieh: Basically, yes. If someone sells sex for money, then she’s a whore, she’s not just easy. […] A whore [...] we know, is socially much lower than someone who is just easy. An easy woman is someone who has a good time, sleeps around—she’s, like, cool—but someone who does it for the money is already a prostitute—that’s, er, way lower.

Labeling WPS as morally flawed supports the radical feminist perspective (Jeffreys, 1997) that views the whore stigma as a central symbolic resource (Shadnam, 2020) in shaping the moral identity of MPWS today. Moreover, Arieh and other participants embody the radical feminist assertion that any moral failings related to sex trading are ultimately attributed to WPS, leaving MPWS unscathed. Thus, they firmly rejected the notion that purchasing sex was wrong. Sometimes, even extreme experiences of critical moments*,* that were acknowledged as such by the participants, triggered no moral breakdown for them*.* Sefi, for example, described his interaction with one WPS who literally called him out on his morally damaged identity:

After I finished one (sexual) act, I started talking to her, sort of small talk. She said something like “You are not a good person, you are a bad person. By coming to me, you are kind of hurting the people you go to, or me.” […] Um, like she was trying to tell me, she put a mirror up to me, like “You’re not here to consume a service, you know like at the supermarket when you buy something and its yours.” By the way, I was with her, you know, very gentle and sensitive, I wasn’t aggressive and I didn’t do something she didn’t want. […] And like, she didn’t say any more. But at some point, I realized that, like, it did bother me, what she said did bother me. When I think about it, er, I don’t know if I hurt her. Personally, I don’t think that I hurt her, but like, that’s what she said—I don’t know. [Sefi]

Sefi experienced a critical moment when the woman whom he had paid for sex described him as “a bad man”—it brought him face-to-face with the hurt he may have inflicted on her, and perhaps on other WPS, like she suggested. However, despite this obvious and acknowledged critical moment (Boltanski & Thévenot, 1999), Sefi did not engage in moral reflexivity (Shadnam, 2020), but rather quickly dismissed the accusations directed at him, and stressed that his conscience is clear. In this sense, Sefi’s example suggests that sincere moments of moral reflexivity do not necessarily result in a reconstruction of one’s moral self. Rather, sometimes such moments are followed by a continuation of one’s established course of action:

I get it that if you’re in the industry, there’s a mental price to pay. Maybe not all the girls understand that right away, and so on, but like—sorry for being honest about it—it’s like, I personally have no problem with it. It’s like, let’s say that I’m even aware of the price that she pays when she meets me, and that it might leave some scars here and there. […] There’s no way for her to do it without being hurt, you know what I’m sayin’? It’s not like, I meet her for a cup of coffee, I interview her, and then go. We get together to have sex, so I can touch her and so on […] If, like, every woman in prostitution gets hurt, then, er—I’m sorry. It’s like, what’s the expression—“Your choice, your… (I-don’t-know-what)”? Like, it’s your choice, so deal with it. […] Like, I personally don’t feel bad about it—my conscience is clean. [Sefi]

We presume that by suggesting that any issues with sex trading are for the WPS to “deal with” and asserting that his “conscience is clean,” Sefi effectively shifts the moral responsibility onto those who sell sex.

Similarly, the following dialog with Arieh demonstrates how he insisted that his moral self was irreproachable, even when he was somewhat provoked by the interviewer to stimulate moral reflexivity. Arieh described his shock when he realized that the woman he was paying was texting on her phone during the sexual act:

Arieh: She did it with complete indifference. Some girls are like that—it just doesn’t interest them. [..] A kind of indifference. She was texting the whole time [pause]. You’re literally inside her, and she’s texting other people at the same time—I dunno, maybe she was talking with her girlfriends [guffaws]—I don’t know. I don’t know what to tell you, it’s like, “Are you’re that…???” That’s already like a machine. She’s a machine [pause]

Interviewer: And how is it for you?

Arieh: It doesn’t feel good—it doesn’t feel good when someone’s like a machine and, like, while you are, er… she’s texting. That’s a machine. I was with this girl that [pause], during the act I’m just looking at her and she’s with her smartphone on the bed, and texting, while I was on top of her. It was nuts. [...] I realize that they do this for the money, so maybe this way they’re, like, trying to… to…—you know, suppress it, forget about it, I dunno. I don’t know how to explain it, what they do [sighs]. Look, some of them, you can see that—they’re having fun, you can feel it. They play along, fully. But texting the whole time, it’s like she turned into a machine.

Interviewer: But what I can’t understand is, how do you feel about the fact that she’s a machine. How do you understand it? How do you see it?

Arieh: I understand that, you know, she’s able to keep her job totally separate from her life. And so she does.

Interviewer: Why does she need to make this separation?

Arieh: Because apparently women can do that—she, er, needs to, and can make that separation.

Interviewer: Why?

Arieh [pauses]: Good question. Because some people are able to do that.

Interviewer: How do you feel after being with someone like her?

Arieh: You’d feel like crap. You’d feel like crap. It’s not a good feeling when you’re [sighs] on top of her and she’s texting at the same time, it like [pauses], it’s like you’re with a machine.

Interviewer: Does it say something about you—that you’re with a machine?

Arieh: No—it doesn’t say anything about me [in an aggressive and dismissive tone]. It says something about her, not about me.

Interviewer: So it says nothing about you?

Arieh: No no no no no no.

Interviewer: It just feels bad.

Arieh: Totally bad. Listen, I see that you have lots of questions, but I really have to go.

While Arieh acknowledged that the woman’s decision to text during the sexual act might indicate her distress and dissociation—in his words, “trying to […] forget about it”—he was either unable or unwilling to morally reflect on his role as the man whose actions she was trying to ignore. The interviewer’s prolonged, yet somewhat futile, attempt to provoke real-time moral reflection in Arieh underscores the influence of radical feminist discourse on shaping the interview interactions and, consequently, the moral identity of the participants. Despite efforts to maintain a nonjudgmental atmosphere and foster a complex understanding of MPWS, the interviewer unconsciously criticized Arieh for not accepting moral responsibility for the woman’s need to dissociate and turn herself into “a machine.” This situation underscores the profound impact of the radical feminist perspective not only on the participants’ self-presentations, but also on the interview environment and interactions, thus significantly influencing the findings.

The interviewer’s active attempts to provoke moral reflection led to emotional defensiveness from Arieh, as the interview setting did not provide a conducive environment for engaging in moral reflexivity. In fact, Arieh became so frustrated that he abruptly ended the interview. This incident illustrates the significant challenges of engaging in moral reflexivity when stigma is present, as it almost automatically elicits a defensive response. It therefore raises questions about the effectiveness of a radical feminist approach that shames MPWS, challenging its ability to motivate them to engage in moral reflexivity, a topic we will explore further later.

Finally, we suggest that the examples above indicate that in the hidden context of SFP, some MPWS lack the symbolic resources (Shadnam, 2020) needed to engage in moral reflexivity. In other words, they lack the requisite language, identities and perhaps even the justification that are needed as symbolic tools for moral reflexivity and identity construction. We therefore argue that a highly stigmatizing and usually hidden social context (such as the sociocultural context of SPF) may result in the individuals involved having a poor arsenal of symbolic resources (Shadnam, 2020) to construct their moral self. This highlights the importance of contextualizing moral reflexivity and the construction of moral self within the wider sociocultural context (Shadnam, 2015).

### Presenting a Reflexive Agonizing Moral Self

The second process of moral self construction that we identified relates to self-reflexivity as a presentation of a moral self. We noticed that for some of the participants, contemplating their own morality was not merely a part of their process of reconstructing a new sense of self, but had become the very core of their moral being. For them, the critical moment (Boltanski & Thévenot, 1999) turned into a protracted critical awareness that they were unable to get past:

It’s hard for me to take part in… an abusive industry. It’s an industry of sexual abuse. Thats what it is, ultimately. No matter how hard we try to sugar-coat it, or to describe it as “free-market services” or a “provider-client” kind of relationship, it’s still sexual abuse. I don’t believe that any woman honestly and truly wants to do this [sell sex]—especially when she has other options of making a living the same level of income. I really don’t think so. That’s it, it’s not easy or simple […] it’s a daily struggle [for me]. It’s… it’s about being a part of an entire system of abuse, and no matter how hard you try to turn it around, it’s difficult to see yourself as part of that system. [Arik]

Arik and other participants discussed the moral conflict they were struggling with, thoroughly and at length. They described feelings of shame and regret, yet kept paying for sex. They were at once satisfied and unsatisfied with their behavior, but above all, were in a constant state of moral reflexivity (Shadnam, 2020) over the harm they might be causing the women they had paid:

We text on WhatsApp, sometimes we call each other, talk, and take an interest in one another. She is sort of a dear friend, a confidant. She is someone I can, uh, I feel free to share what’s going on in my life with. When I’m feeling bad, when I’m feeling good, when she’s feeling bad, when she’s feeling good. It’s a sort of, a sort of paid lover, let’s call it that […] I try to look at myself as someone who is supporting her not just for sex, but rather as someone who is supporting her to get along in life—and we just happen to be doing it [having sex] as well. […] Maybe I’m saying this just to clear my conscience, because I don’t feel comfortable with it. I mean, I keep wondering, and I also asked her, I told her “Let’s say that you had everything going for you, and you had a decent income and you could take care of yourself—would you still do this?” And she said no, she wouldn’t. Which means that she’s doing it because she has no other choice […] Like I said, I don’t feel comfortable doing this, but I try to look at it as though I’m helping a friend, rather than something else. Am I really persuading myself? No. But I’m trying to. [Oron]

Oron was troubled by the realization that the woman he pays might only be engaging with him for the money. The internal dynamic he described reflects the tension between men’s desire to control access to women and their simultaneous wish for women to genuinely desire them (Gunnarsson & Strid, 2022). Although he saw the woman as a dear friend or even a lover and enjoyed their interactions, Oron questioned the authenticity of their relationship. Despite his doubts, he could not bring himself to end the relationship. This highlights how the illusion of mutual authenticity (Sanders, 2008) can become entangled with moral dilemmas, suggesting that the line between authentic and fabricated mutual feelings is often blurred.

Unable to untangle the moral conflict, Oron and other participants appear to have simply succumbed to the moral morass in which they are struggling. They have accepted it. We suggest that since they were unable to find a moral solution that would ease their guilt yet allow them to keep paying for sex, some of the participants adopted an agonized moral being, instead:

Even after I have a pleasurable sexual interaction, or an emotionally satisfying experience, I still feel a pang of regret. I mean, there are different voices inside me, and it’s a very tangled situation for me, especially since I’m aware of the ramifications for those who engage in prostitution. And because I’m a religious man, and understand the outcomes that my actions have for me, and how it directly hurts me. It’s like taking a knife and ever-so-slowly cutting myself [demonstrates cutting his own thigh, as he speaks]. […] I pay for sex and tell myself that it is despicable, and that it should be avoided—but I keep doing it. What’s my excuse? I have no excuse. I know this is who I am, and I’m ashamed of myself. Even when I go there, or when I’m done, or afterward, I’m ashamed of myself. [Tamir]

Tamir is deeply troubled by the distress his actions cause to WPS. As a religious man, his moral breakdown (Zigon, 2007) is further exacerbated by the harsh religious condemnation portraying Jewish MWPS as sinful (Levy, 2020). This highlights the simultaneous impact of various symbolic resources that shape the moral norms with which MWPS must grapple. In the current Israeli context, it is not only feminist criticism that depicts MWPS as exploiters but also religious condemnation that labels them as “failed” Jewish men.

Despite the profound and multifaceted anguish, Oron and Tamir feel trapped in this situation. We argue that for them and for other participants, moral reflexivity has become the very essence of their moral identity. Moreover, since paying for sex is a hidden phenomenon that is usually kept secret (Kong, 2016), presenting a reflexive moral self is possibly what motivated some of the participants to take part in the study. Perhaps they sought an opportunity to alleviate their internal, concealed moral struggle in the hope of achieving even temporary relief. Specifically, some participants might have hoped that being shamed for their engagement in SFP would serve as a “punishment” that mitigates their sense of guilt. Thus, for example, at the start of his interview, Gershon stated that he wants to be criticized for his actions as a man who pays for sex:

You probably have your own didactic or educational purpose, or whatever—and that’s fine. I can say that you can really feel very comfortable—er, you mentioned insights—but I rarely get to talk about it, obviously, with other people. So, I, I usually don’t hear criticism. […] so don’t, er, I came here today for the purpose of that you can feel free to criticize me on this matter—it’s totally fine. [Gershon]

After presenting his agonizing and painful moral self narrative, toward the end of the interview Gershon asked once again to be criticized, and even yelled at:

Gershon: But er, I’ve said enough, I want you to yell a bit. No, I, I, [clicks his tongue]. I mean, yes, I want to hear your criticism.

Interviewer: I feel like you want me to, as it were—you came to get “a tongue-lashing.”

Gershon: […] Er yes, I want to hear what you think about this.

Being invited to participate in the study appeared to be a critical moment for Gershon, triggering a process of moral reflexivity*,* followed by a request to be criticized and shamed. Unlike previous instances where participants rejected any moral condemnation, particularly during the interviews, Gershon saw the invitation to be criticized as an opportunity to reveal an ongoing self-reflexive moral self that he typically keeps hidden. We suggest that, perhaps as an Ultra-orthodox man, the interview acted as a confessional setting (Sanders, 2008), allowing Gershon to articulate his deepest and most distressing moral conflicts. This illustrates the complex and sometimes contradictory interplay between discursive contexts and interactional settings in shaping moral identities. Furthermore, the interview with Gershon demonstrates the complex nature of moral reflexivity, whereby individuals do not necessarily complete the full circle of external trigger—self-reflection—identity reconstruction. Rather, these phases may overlap and coexist in the individual’s attempt to construct and maintain a moral identity.

### Constructing a Moral Self Through Identity Fragmentation

The final process that we identified is that of constructing a moral self through identity fragmentation. We found that for some of the participants, the moral breakdown (Zigon, 2007) was so profound that the only way to manage it and recover from it was by fragmenting their identity. Through the process of moral reflexivity (Shadnam, 2020), these participants re-invented themselves as fragmented moral beings by detaching the immoral, paying for sex, self from what they called their “true self.” This fragmentation was evident in their actual SFP experiences and in their retrospective contemplation of their past engagements with the sex industry.

For some of the participants, the moral breakdown (Zigon, 2007) was inherently linked to their involvement in the sex industry. For Jacob, for example, the very first SFE experience involved a severe breakdown which he struggled to recover from: realizing that the woman he had paid for sex was a mother with children triggered a critical moment (Boltanski & Thévenot, 1999) in him, after which he could no longer pretend or fantasize about the relationship with her being exclusive, mutual, or romantic:

She urged me to sneak out and get out of there as soon as possible: she wanted to get back, and maybe there’d be another client. I gave her more money than she asked for, and she, eh, really thanked me—she was surprised. She said something about her children—I can’t really remember—and that now they would have something to eat—a detail that obviously shattered every bit of fantasy there [guffaws] about exclusivity, which clearly wasn’t the case. Umm, providing for children. A mother. Er, something about her being a mother, my stimulation kind of tanked [sighs]. It’s like, I could have thrown up. I’m not someone who usually throws up, but like, I was disgusted: What have I done? How low can I go? It mixed up with the [sexual] excitement and release, and felt horrible, like altogether. […] Er, [I felt] that I had to get away, as fast as I could. So I went back to the [the army] base and took a shower, and obviously it wasn’t like that, but I felt like it was two hours long, in which I cried, nonstop. [Jacob]

After that experience, Jacob described moral breakdown as being an integral part of his ensuing experiences in SFP (which went on for over two decades). For him, the disillusionment from the fantasy of authentic mutuality in paid-for sexual encounters (Sanders, 2008) entailed an ongoing moral crisis. Unlike the participants who embraced this moral crisis and presented an agonized moral self (as described earlier), Jacob and other participants distanced themselves from their paying-for-sex-self while silencing moral judgments, in an effort to maintain a sense of self-worth. These participants compartmentalized their personas as MPWS to resist stigma attached to sex trading and defend their “true” self-identity as “good” men (Curtis et al., 2021):

You have these voices inside that scream at you that you shouldn’t be doing this, and it’s wrong, and “What the hell are you doing?” And then you have a sexual urge that, in order to satisfy it, to relieve it, all the other voices need to be silenced […] It’s like a kind of pendulum that goes from forbidding sexuality to denial […] of the forbidden and ignoring the too-rigid conscience—and then I swing to the other side, and I’m like, “Fuck it!,” like, “I’m not in the Moral Zone anymore, I’m doing what… I’m rebelling, I’m doing whatever I want!”—without considering the other person […] It’s a dissociative experience. If I’m within a sexual quest, er, there are many parts of my personality that are actually frozen, silenced—they’re not there, er, there is no longer a consideration of right or wrong. [Jacob]

Participants described the fragmentation of their identity as an uncontrolled mechanism that helped preserve their self-worth during the prolonged moral crisis they experienced. Referring to their experience in SFP as dissociative symbolizes the centrality of radical feminist concepts of sexual violence in shaping the sex trade discourse. Suggesting that MPWS struggle with dissociative disorders might illustrate how ideas, notions, and discourses may unexpectedly migrate across fields. Mentioning dissociation in this particular context and distancing the paying-for-sex-self—be it uncontrolled processes or self-aware behaviors—enabled Jacob and other participants to navigate the heavy moral burden that resulted from their actions. It might also suggest that discussing sex trading in terms of trauma allows some MPWS to express their own pains while maintaining a sense of positive self-perception:

The inner conflict is terrible, it’s terrible at that point. Just horrendous. There are times when these two characters are completely detached from one another: there is Moshe who can stand and deliver a lesson [on the Torah] to everyone—and then, er, the other Moshe absolutely doesn’t exist. And then, of course, when I go out to Tel Aviv—and for me Tel Aviv was like Sodom, you know—the kind of place where, once you go through the gates of the city, you become a different person. […] and from that moment on, the let’s say the “good” voices in your head [chuckles] disappear […]. This inner conflict [...] hurts, there are moments when it’s really painful, really really painful […]. It’s a kind of split—like, at certain moments, Dr. Jekyll and Mr. Hyde. It’s like one character doesn’t meet the other. Again—it’s not that he doesn’t know what the other one did last night, if you were to remind me, I’d remember very quickly, but you just don’t think about it. [Moshe]

Once again, the progression from the critical moments—to moral reflexivity, to reconstruction of a moral identity—appears to be a nonlinear one. Rather, we suggest that Shadnam’s model should be conceptualized as a messy process that includes acute and prolonged moral breakdowns that may trigger constant attempts to reconstruct and maintain a sense of self-worth and moral self. In other words, we argue that Shadnam’s model should be viewed as a heuristic conceptual framework for understanding the formation of moral identity. Thus, for example, the prolonged moral crisis and reconstruction of the moral self coexisted and overlapped in the narrative of several participants in the present study.

Interestingly, after ending his habit of paying for sex and engaging in long-term processes of moral reflection (mainly through therapy), Jacob described how he reconstructed his moral self by reconciling all his identity fragments into an integrated whole:

[When I started therapy] this shield that separated the two characters, the two personalities, started to crumble. And then came depression, and self-loathing, er, and tones of regret—right to this day I feel huge regret, er, a will to fix things and make amends as much as possible, and it’s not always possible. […] But today I can feel myself without all the fragmented pieces, but as a whole person. The person who is sitting with you here today is the same private Jacob who recovered from hyper-sexuality and [...] from paying for sex—it’s like, all of those things have come together today. It may be more comfortable, or less, but they’re sitting together around the same, er, the same table, living under the same roof, and not in different apartments.

The resolved sense of moral self became accessible to Jacob only after he ceased paying for sex. We therefore suggest that the symbolic resources available for one’s moral reflexivity are sometimes contingent upon whether one continues to engage in condemned behaviors. Moreover, Jacob’s description points to the crucial role that the presence of others can play in the formation of individuals’ sense of moral self. In therapy, Jacob’s moral reflexivity resulted in a reconciled and less conflicted sense of moral self—one that he was unable to achieve on his own. This highlights the importance of the context of the process of forming a moral self—not only in the broader sense of social norms, but as it relates to the particular aspects of time, space and others, within which one reflects and interprets one’s behavior (Hitlin & Vaisey, 2013; Shadnam, 2015).

## Discussion

The findings of this study provide new and profound insights into how Israeli MPWS construct and maintain a moral identity, using the conceptual framework of *moral reflexivity* (Shadnam, 2020). Through a flexible and heuristic use of this multidimensional theoretical concept, we identified three main processes of moral identity construction by MPWS: Maintaining an intact moral self by resisting the moral conflicts, Presenting a reflexive agonizing moral self, and Constructing a moral self through identity fragmentation. We further showed that the dimensions of the moral reflexivity process (such as external irritation—self-reflection—identity reconstruction) may overlap, coexist and circulate in a messy back-and-forth fashion, creating complex dynamics through which MPWS negotiate their moral self and contemplate the social stigma that paints them as morally flawed.

The findings demonstrate the embedded nature of the processes of constructing a moral identity within given social contexts, in relation to wider social constructs and to specific interactional ones. We found that the participants constructed their moral identity mostly within the framework of radical feminist discourse, which views sex trading as a form of sexual violence. This perspective is particularly relevant in the Israeli sociolegal context that implements this discourse by criminalizing MPWS. Additionally, the religious condemnation of Jewish MPWS and the dynamics of being interviewed by a woman presumed to be judgmental of their actions also significantly impacted the participants’ moral identity. However, it appears that the participants interpreted these discursive and interactional contexts in varied and sometimes contradictory ways when constructing their moral selves.

For example, while some participants grappled with their exploitative actions to maintain a moral self, others rejected radical feminist accusations that depicted them as villains. Furthermore, for some participants, the mere anticipation of being stigmatized and criticized by the interviewer triggered the moral reflexive process, as they saw the interview as a rare opportunity to express a moral reflection they were already engaged in. For other participants, this anticipation had the opposite effect—prompting them to vociferously reject the idea that moral reflexivity is needed, and to reject the opportunity to engage in it within the interview. This complexity underscores the need for further development of the theoretical concept of identity, especially moral identity, in the context of sex trading and MPWS. The conceptual framework of identity reveals the intricate interplay between social constructs, interactional settings, and personal attributes and how this interplay manifests in individuals’ daily behaviors. Specifically, understanding the multiple dimensions of how MPWS make sense of their morally condemned behaviors illustrates that morality is not a static or essentialist entity. Instead, it is a dynamic structure that continuously evolves (Hitlin, 2023).

The changing impact of the contextual and interactional settings in shaping the scene of moral reflexivity manifests in the spectrum of mechanisms employed by the participants to construct their moral identity. This suggests that a different type of interactions (such as with a male interviewer, perhaps, or in an online forum for MPWS) may have resulted in different processes of moral identity construction. It is also possible that in societies where MPWS are less stigmatized, by feminist and/or conservative discourses, processes of moral reflexivity may not be triggered at all. Thus—in line with Shadnam’s call to “have a theoretical framework that systematically guides us toward specifying the important dimensions of the scene of moral reflexivity” (Shadnam, 2020)—we argue that the interactional, situational and discursive contexts are crucial aspects that must be explored as part of the study of moral reflexivity.

We also found a great variety in the types of critical moments that triggered the participants’ moral reflexivity processes. Some of the participants described spontaneous critical moments that occurred unexpectedly during their encounters with WPS and spark an inner moral reflection within themselves, while others defended themselves against the interviewer’s attempts to initiate such moments. Indeed, several participants described how certain “eye-opening” critical moments transformed into prolonged experiences of self-doubt. The variety of critical moments and the intense moral reflection that sometimes followed them suggest that merely confronting MPWS with their wrongdoings and the potential harm they may cause, does not always lead to moral reflection. It therefore raises questions about the potential efficacy of such confrontations when applied as part of social-educational interventions aimed at reducing sex consumption (such as “John Schools”).

Nonetheless, the findings do suggest that some MPWS do yearn for “safe spaces” in which they can reflect, ponder, and doubt themselves. These participants recounted that meaningful and emotional processes of moral reflexivity occurred within therapeutic settings in which they did not feel judged, criticized, or stigmatized. We propose that such therapeutic spaces would allow MPWS to consider the ramifications that paying for sex may have for themselves, and most importantly, to be free of shaming and moral judgment. Paradoxically, the absence of such moral judgment may be more likely to prompt moral reflexivity among MPWS.

The findings demonstrate the complexity of moral identity that cannot be reduced to binary discussions of good or bad, and right or wrong. The processes of moral reflexivity that we identified were usually characterized by doubt, hesitation, and contradicting experiences and perceptions. Moral identity is therefore not an essentialist entity, but rather a context-bound structure that constantly changes. Its value is continuously determined by both broad and situational circumstances that define what is considered acceptable or unacceptable. Thus, the findings contribute to the conceptualization of moral identity not as a pre-discursive entity; but rather an ever-changing sense of self that cannot exist outside of a social context. Accordingly, we align with the argument made by others that the goal of a study of morality should be directed at promoting an ecological understanding of *how* morality is shaped by various societal forces, rather than establishing *what* is a moral act, being, or identity (Hitlin, 2021). In the context of MPWS, the heated debate over the morality of MPWS and the sex industry, and their binary categorization as either immoral abusers or normative moral men, should be replaced with a complex and nuanced understanding of MPWS.

Morality and moral identities are highly relevant to the study of MPWS and the sex industry, and we argue that moral issues should be further explored within a variety of sex industry settings. In any event, we call for a morally aware or morally sensitive approach in studies and interventions in this field. The objective should not be to determine which behaviors are moral, or who is morally flawed. Rather, adopting a morally sensitive approach means that researchers and policy makers should be aware of moral issues, moral questions and moral processes; treat morality as a social-context-dependent attribute that is highly relevant to the field of the sex industry; and attempt to promote a multifaceted understanding of how moral identities are shaped in various contexts of the sex industry, and through diverse research methods and intervention models.

### Limitations

This study explored the moral identity of 23 Israeli MPWS. While the sample was heterogeneous in terms of marital status and age, it was homogeneous in terms of religious background as all of the participants identified as Jewish. Religiosity serves as meaningful symbolic resources in constructing moral identity and therefore it affects and limits the transferability of the findings. In line with the premise of constructivist qualitative research, in any given study, only partial and context-bound understanding is possible (Charmaz, 2014). Hence, this study underlines the need for further investigation of the moral identity of MPWS, particularly of differing religious background, to gain a better understanding of its layered facets. Furthermore, the role of specific personal characteristics, such as marital status, in shaping the moral identity of MPWS should also be further explored.

Finally, this study is pioneering in its application of the conceptual framework of moral identity to the research of MPWS. However, many aspects concerning the moral considerations of MPWS remain unexplored. For instance, it is crucial to investigate why MPWS continue to pay for sex even after experiencing moral conflict or while grappling with an agonizing moral self. It was beyond the scope of this research to examine how moral identity interacts with other identities that MPWS hold and negotiate. We cautiously suggest that exploring the interplay between the moral identities of MPWS and their masculinity for example could enhance our understanding of their decision-making processes when engaging in SFP and their subsequent actions. We encourage other researchers to pursue this and other lines of inquiry concerning the morality of MPWS.

## Footnotes

1 In the Israeli-Jewish context, prostitution (as opposed to sex trading) is the most common term.
